# Draft genome sequences of 148 *Klebsiella pneumoniae* species complex members from bovine and human hosts

**DOI:** 10.1128/mra.00132-24

**Published:** 2024-06-12

**Authors:** Bridget O’Brien, Alla Yushchenko, Jinha Suh, Dongyun Jung, Zhangbin Cai, Ngoc Sang Nguyen, Makeda Semret, Simon Dufour, Jennifer Ronholm

**Affiliations:** 1 Faculty of Agricultural and Environmental Sciences, Macdonald Campus, McGill University, Montréal, Québec, Canada; 2 Mastitis Network, Saint-Hyacinthe, Québec, Canada; 3 Regroupement FRQNT Op+Lait, Saint-Hyacinthe, Québec, Canada; 4 McGill University Health Centre (Infectious Diseases and Medical Microbiology), Montréal, Québec, Canada; 5 Faculté de Médecine Vétérinaire, Université de Montréal, Saint-Hyacinthe, Québec, Canada; Wellesley College Department of Biological Sciences, Wellesley, Massachusetts, USA

**Keywords:** *Klebsiella*, Bovine Mastitis, Klebsiella pneumoniae sensu stricto

## Abstract

*Klebsiella pneumoniae* species complex members, particularly *K. pnemoniae sensu stricto*, are common bovine clinical mastitis pathogens and often the cause of hospital- and community-acquired infections in humans. Here, we present 148 draft genome assemblies and annotations of *K. pneumoniae* species complex members from bovine and human hosts in Canada.

## ANNOUNCEMENT

Comprising *Klebsiella pneumoniae sensu stricto*, *Klebsiella quasipneumoniae* subsp. *quasipneumoniae*, *K. quasipneumoniae* subsp. *similipneumoniae*, *Klebsiella variicola* subsp. *variicola*, and *K. variicola* subsp. *tropica*, members of the *K. pneumoniae* species complex (KpSC) cause infections in both humans and dairy cattle (1, 2). *K. pneumoniae sensu stricto*, herein referred to as *K. pneumoniae*, is the predominant KpSC isolate in bovine mastitis and human infections, such as pneumonia, bacteremia, and urinary sepsis (2–7). Relatively few KpSC isolates from Canada have been studied, making publicly available KpSC genomes from Canada a valuable resource for further research.

Here, we present draft genome sequences of 148 KpSC isolates from human (*n* = 78) and bovine (*n* = 70) hosts. *K. pneumoniae sensu strico* represented 86% of KpSC isolates (bovine, *n* = 65; human *n* = 63), *K. variicola* subsp. *variicola* represented 8% (bovine, *n* = 2; human, *n* = 10), and both *K. quasipneumoniae* subsp. *quasipneumoniae* (bovine, *n* = 3; human, *n* = 1) and *K. quasipneumoniae* subsp. *similipneumoniae* (human, *n* = 4) represented 3%.

Bovine isolates were obtained from the Mastitis Pathogen Culture Collection (8). Briefly, these isolates were confirmed as *Klebsiella* spp. using biochemical tests (lactose positive; indole, citrate, and oxidase negative) after plating 10 µL of raw milk on Columbia agar with 5% sheep blood and MacConkey agar. In accordance with the institutional ethical guidelines at McGill University and the Declaration of Helsinki, up to 10 mL of blood from patients at the McGill University Health Centre was inoculated directly into BACT/ALERT aerobic and anaerobic blood culture bottles (BioMérieux) which contain 40 mL tryptic soy broth (TSB), and placed on the BacT/ALERT automated platform at 35°C for incubation. Bottles flagged as culture-positive were subcultured onto blood and MacConkey agar using one drop of the incubated blood. Following growth, pure colonies were identified as *K. pneumoniae* using Matrix-assisted laser desorption ionization–time-of-flight mass spectrometry (MALDI-TOF MS), as described previously (9). Each isolate, stored in a stock solution at −80°C, was thawed, streaked onto tryptic soy agar (TSA), and incubated overnight at 37°C. One colony was picked from the TSA plate, added into 5 mL of TSB, and grown overnight at 37°C under shaking conditions (~200 rpm). DNA was extracted from liquid cultures using DNeasy Ultraclean Microbial kit following manufacturer’s instructions (Qiagen, USA). DNA quality was assessed using the NanoDrop 2,000 spectrophotometer (ThermoScientific), and the concentration was determined using Quant-IT dsDNA High-Sensitivity assay (Invitrogen, USA). Samples with A260/A280 ratio < 1.8 and A260/A230 ratio between 1.8 and 2.2 were included in library preparation using Nextera DNA Flex Prep kit following manufacturer’s instructions (Illumina, USA). Libraries were sequenced using the MiSeq benchtop sequencer (Illumina) and the MiSeq Reagent Kit v3 (2 × 300 bp), yielding a total of 153,632,994 reads. Reads were assembled *de novo* using ProkaryoteAssembly (v. 0.1.6) (https://github.com/BFSSI-Bioinformatics-Lab/ProkaryoteAssembly) with default parameters, apart from the trimming step in which low-quality sequences with Q-score < 20 were filtered out. Following assembly, contigs with fewer than 1,000 bp were removed. Quality of final assemblies was assessed with QualiMap (v. 2.2.2) (10). Species identification was completed using Kleborate (v. 2.4.0) (11). Gene annotations and predictions were performed using NCBI Prokaryotic Genome Annotation Pipeline (v. 6.6) upon submission to GenBank (Table 1) (12).

**TABLE 1:** Genome quality and predications of *K. pneumoniae sensu strico*, *K. variicola* subsp. *variicola*, *K. quasipneumoniae* subsp. *quasipneumoniae*, and *K. quasipneumoniae* subsp. *similipneumoniae* assemblies

Isolate identification number/name	Host	Species	BioSample accession no.	GenBank accession no.	SRA accession no.^*a*^	Draft genome size (bp)	Coverage (X)	G + C content (%)	No. of contigs	N50 (bp)	No. of CDS^*b*^	No. of RNAs
10106020	Bovine	*K. pneumoniae sensu strico*	SAMN38811387	JAXSFJ000000000	SRR27199061	5,425,478	30	56.43	115	103,101	5,373	92
10106228	Bovine	*K. pneumoniae sensu strico*	SAMN38811388	JAXSFI000000000	SRR27199060	5,228,123	46	56.84	87	159,343	5,147	92
10108161	Bovine	*K. pneumoniae sensu strico*	SAMN38811389	JAXSFH000000000	SRR27198969	5,232,226	35	56.69	87	148,395	5,155	92
10108963	Bovine	*K. pneumoniae sensu strico*	SAMN38811390	JAXSFG000000000	SRR27198958	5,226,237	34	56.84	101	119,473	5,154	87
10116548	Bovine	*K. pneumoniae sensu strico*	SAMN38811391	JAXSFF000000000	SRR27198947	5,427,354	32	56.32	104	141,038	5,366	94
10116616	Bovine	*K. pneumoniae sensu strico*	SAMN38811392	JAXSFE000000000	SRR27198936	5,371,671	18	56.37	292	40,642	5,449	74
10116623	Bovine	*K. pneumoniae sensu strico*	SAMN38811393	JAXSFD000000000	SRR27199021	5,423,477	26	56.46	113	114,031	5,369	90
10116692	Bovine	*K. pneumoniae sensu strico*	SAMN38811394	JAXSFC000000000	SRR27199010	5,428,749	33	56.26	101	118,039	5,365	92
10116739	Bovine	*K. pneumoniae sensu strico*	SAMN38811395	JAXSFB000000000	SRR27198999	5,435,163	46	56.36	95	148,395	5,373	93
10201992	Bovine	*K. pneumoniae sensu strico*	SAMN38811396	JAXSFA000000000	SRR27198924	5,224,805	28	56.7	122	119,474	5,168	95
10202968	Bovine	*K. pneumoniae sensu strico*	SAMN38811397	JAXSEZ000000000	SRR27199059	5,230,909	36	56.6	90	159,221	5,150	90
10214985	Bovine	*K. pneumoniae sensu strico*	SAMN38811398	JAXSEY000000000	SRR27199048	5,385,679	39	56.59	26	798,846	5,200	94
10216484	Bovine	*K. pneumoniae sensu strico*	SAMN38811399	JAXSEX000000000	SRR27199037	5,237,069	39	56.64	88	159,221	5,158	90
10300398	Bovine	*K. pneumoniae sensu strico*	SAMN38811400	JAXSEW000000000	SRR27198994	5,658,740	29	56.14	79	182,860	5,563	86
10300862	Bovine	*K. pneumoniae sensu strico*	SAMN38811401	JAXSEV000000000	SRR27198983	5,370,163	36	56.51	146	119,659	5,353	92
10302521	Bovine	*K. pneumoniae sensu strico*	SAMN38811402	JAXSEU000000000	SRR27198974	5,403,094	57	56.83	50	266,010	5,266	97
10309735	Bovine	*K. pneumoniae sensu strico*	SAMN38811403	JAXSET000000000	SRR27198973	5,268,042	48	56.83	88	159,343	5,189	94
10309742	Bovine	*K. pneumoniae sensu strico*	SAMN38811404	JAXSES000000000	SRR27198972	5,463,147	59	56.72	40	366,197	5,298	90
10310236	Bovine	*K. pneumoniae sensu strico*	SAMN38811405	JAXSER000000000	SRR27198971	5,365,745	15	56.75	309	32,624	5,413	59
10310267	Bovine	*K. pneumoniae sensu strico*	SAMN38811406	JAXSEQ000000000	SRR27198970	5,277,068	40	56.59	89	159,343	5,209	90
10400807	Bovine	*K. pneumoniae sensu strico*	SAMN38811407	JAXSEP000000000	SRR27198968	5,297,921	59	56.89	39	421,036	5,156	95
10402672	Bovine	*K. pneumoniae sensu strico*	SAMN38811408	JAXSEO000000000	SRR27198967	5,292,939	40	56.89	23	506,305	5,164	101
10413500	Bovine	*K. pneumoniae sensu strico*	SAMN38811409	JAXSEN000000000	SRR27198966	5,387,201	26	56.23	119	97,898	5,334	91
10415139	Bovine	*K. pneumoniae sensu strico*	SAMN38811410	JAXSEM000000000	SRR27198965	5,426,595	25	56.56	105	180,680	5,266	94
10415269	Bovine	*K. pneumoniae sensu strico*	SAMN38811411	JAXSEL000000000	SRR27198964	5,481,433	77	56.57	44	297,708	5,355	93
10417423	Bovine	*K. pneumoniae sensu strico*	SAMN38811412	JAXSEK000000000	SRR27198963	5,424,186	39	56.26	96	142,713	5,357	94
10417447	Bovine	*K. pneumoniae sensu strico*	SAMN38811413	JAXSEJ000000000	SRR27198962	5,472,868	28	56.38	60	239,632	5,344	101
10417843	Bovine	*K. pneumoniae sensu strico*	SAMN38811414	JAXSEI000000000	SRR27198961	5,383,914	45	56.02	91	159,343	5,304	93
10418109	Bovine	*K. pneumoniae sensu strico*	SAMN38811415	JAXSEH000000000	SRR27198960	5,723,621	49	56.05	64	204,589	5,613	97
10418338	Bovine	*K. pneumoniae sensu strico*	SAMN38811416	JAXSEG000000000	SRR27198959	5,713,685	26	56.04	85	158,630	5,648	97
11003939	Bovine	*K. pneumoniae sensu strico*	SAMN38811417	JAXSEF000000000	SRR27198957	5,457,274	35	56.6	56	381,920	5,278	97
11005407	Bovine	*K. pneumoniae sensu strico*	SAMN38811418	JAXSEE000000000	SRR27198956	5,531,908	34	56.41	51	288,946	5,381	91
11005414	Bovine	*K. pneumoniae sensu strico*	SAMN38811419	JAXSED000000000	SRR27198955	5,530,947	38	56.28	44	375,673	5,373	91
11006091	Bovine	*K. pneumoniae sensu strico*	SAMN38811420	JAXSEC000000000	SRR27198954	5,526,959	41	56.65	51	288,947	5,372	91
11007937	Bovine	*K. pneumoniae sensu strico*	SAMN38811421	JAXSEB000000000	SRR27198953	5,514,281	21	56.23	116	94,010	5,403	91
11007944	Bovine	*K. pneumoniae sensu strico*	SAMN38811422	JAXSEA000000000	SRR27198952	5,534,843	45	56.52	43	355,703	5,378	92
11009085	Bovine	*K. pneumoniae sensu strico*	SAMN38811423	JAXSDZ000000000	SRR27198951	5,507,594	54	56.58	35	375,690	5,341	92
11009146	Bovine	*K. pneumoniae sensu strico*	SAMN38811424	JAXSDY000000000	SRR27198950	5,534,898	53	56.55	42	355,703	5,373	92
11010982	Bovine	*K. pneumoniae sensu strico*	SAMN38811425	JAXSDX000000000	SRR27198949	5,524,841	40	56.38	46	383,384	5,364	90
11113904	Bovine	*K. pneumoniae sensu strico*	SAMN38811426	JAXSDW000000000	SRR27198948	5,480,810	35	56.21	55	346,188	5,377	96
11114840	Bovine	*K. pneumoniae sensu strico*	SAMN38811427	JAXSDV000000000	SRR27198946	5,442,605	49	56.61	47	369,687	5,314	95
11115748	Bovine	*K. pneumoniae sensu strico*	SAMN38811428	JAXSDU000000000	SRR27198945	5,525,882	38	56.42	50	269,910	5,370	90
11708612	Bovine	*K. pneumoniae sensu strico*	SAMN38811429	JAXSDT000000000	SRR27198944	5,617,391	40	56.39	78	214,765	5,502	93
11912781	Bovine	*K. pneumoniae sensu strico*	SAMN38811430	JAXSDS000000000	SRR27198943	5,229,672	36	57.16	29	371,293	5,052	89
21206061	Bovine	*K. pneumoniae sensu strico*	SAMN38811431	JAXSDR000000000	SRR27198942	5,626,718	49	56.03	27	387,075	5,499	94
22513373	Bovine	*K. pneumoniae sensu strico*	SAMN38811432	JAXSDQ000000000	SRR27198941	5,353,554	80	56.61	42	350,392	5,176	94
22713346	Bovine	*K. pneumoniae sensu strico*	SAMN38811433	JAXSDP000000000	SRR27198940	5,380,696	38	56.86	45	304,306	5,238	91
30817180	Bovine	*K. pneumoniae sensu strico*	SAMN38811434	JAXSDO000000000	SRR27198939	5,418,896	30	56.75	51	297,910	5,233	97
40400129	Bovine	*K. pneumoniae sensu strico*	SAMN38811435	JAXSDN000000000	SRR27198938	5,324,373	46	56.42	60	228,832	5,147	91
40611440	Bovine	*K. quasipneumoniae* subsp. *quasipneumoniae*	SAMN38811436	JAXSDM000000000	SRR27198937	5,380,139	31	57.44	63	256,433	5,161	88
40615929	Bovine	*K. variicola* subsp. *variicola*	SAMN38811437	JAXSDL000000000	SRR27198935	5,485,656	40	57.16	24	483,749	5,231	91
40706870	Bovine	*K. pneumoniae sensu strico*	SAMN38811438	JAXSDK000000000	SRR27198934	5,480,982	51	56.65	41	321,408	5,310	98
40706948	Bovine	*K. pneumoniae sensu strico*	SAMN38811439	JAXSDJ000000000	SRR27199029	5,458,436	41	56.66	63	350,390	5,293	83
40706993	Bovine	*K. quasipneumoniae* subsp. *quasipneumoniae*	SAMN38811440	JAXSDI000000000	SRR27199028	5,417,015	34	57.3	54	291,465	5,198	98
40709369	Bovine	*K. pneumoniae sensu strico*	SAMN38811441	JAXSDH000000000	SRR27199027	5,288,319	46	57.01	45	370,742	5,136	91
40713625	Bovine	*K. quasipneumoniae* subsp. *quasipneumoniae*	SAMN38811442	JAXSDG000000000	SRR27199026	5,416,886	29	57.5	67	216,074	5,180	95
40717173	Bovine	*K. pneumoniae sensu strico*	SAMN38811443	JAXSDF000000000	SRR27199025	5,522,691	37	56.58	67	216,982	5,374	101
40718170	Bovine	*K. pneumoniae sensu strico*	SAMN38811444	JAXSDE000000000	SRR27199024	5,490,775	42	56.56	48	475,862	5,316	92
40718231	Bovine	*K. pneumoniae sensu strico*	SAMN38811445	JAXSDD000000000	SRR27199023	5,455,694	41	56.77	50	769,862	5,316	98
40718651	Bovine	*K. variicola* subsp. *variicola*	SAMN38811446	JAXSDC000000000	SRR27199022	5,707,929	38	56.67	45	389,087	5,549	100
40806273	Bovine	*K. pneumoniae sensu strico*	SAMN38811447	JAXSDB000000000	SRR27199020	5,699,707	31	56.36	86	205,377	5,620	89
41006955	Bovine	*K. pneumoniae sensu strico*	SAMN38811448	JAXSDA000000000	SRR27199019	5,279,381	48	56.92	34	360,377	5,150	96
41203163	Bovine	*K. pneumoniae sensu strico*	SAMN38811449	JAXSCZ000000000	SRR27199018	5,315,776	61	56.32	61	229,224	5,133	87
41508275	Bovine	*K. pneumoniae sensu strico*	SAMN38811450	JAXSCY000000000	SRR27199017	5,185,978	52	56.71	80	155,317	4,997	91
41510377	Bovine	*K. pneumoniae sensu strico*	SAMN38811451	JAXSCX000000000	SRR27199016	5,420,290	84	56.71	51	323,739	5,262	93
41518175	Bovine	*K. pneumoniae sensu strico*	SAMN38811452	JAXSCW000000000	SRR27199015	5,350,680	58	56.53	44	255,820	5,239	97
41518533	Bovine	*K. pneumoniae sensu strico*	SAMN38811453	JAXSCV000000000	SRR27199014	5,586,164	87	56.32	72	221,685	5,456	93
41518540	Bovine	*K. pneumoniae sensu strico*	SAMN38811454	JAXSCU000000000	SRR27199013	5,454,922	39	56.28	95	155,030	5,291	89
41518854	Bovine	*K. pneumoniae sensu strico*	SAMN38811455	JAXSCT000000000	SRR27199012	5,450,089	22	56.39	183	71,321	5,419	90
41519141	Bovine	*K. pneumoniae sensu strico*	SAMN38811456	JAXSCS000000000	SRR27199011	5,191,411	60	56.87	83	176,242	5,008	94
HKp1	Human	*K. pneumoniae sensu strico*	SAMN38811457	JAXSCR000000000	SRR27199009	5,438,233	34	56.68	43	231,448	5,258	87
HKp10	Human	*K. pneumoniae sensu strico*	SAMN38811458	JAXSCQ000000000	SRR27199008	5,263,762	43	56.75	37	295,122	5,044	90
HKp11	Human	*K. pneumoniae sensu strico*	SAMN38811459	JAXSCP000000000	SRR27199007	5,274,286	34	56.79	65	212,985	5,071	92
HKp12	Human	*K. pneumoniae sensu strico*	SAMN38811460	JAXSCO000000000	SRR27199006	5,605,874	57	56.63	52	231,035	5,513	100
HKp13	Human	*K. quasipneumoniae* subsp. *similipneumoniae*	SAMN38811461	JAXSCN000000000	SRR27199005	5,568,097	28	56.7	67	329,287	5,429	88
HKp14	Human	*K. pneumoniae sensu strico*	SAMN38811462	JAXSCM000000000	SRR27199004	5,383,514	63	56.61	39	362,892	5,223	96
HKp15	Human	*K. variicola* subsp. *variicola*	SAMN38811463	JAXSCL000000000	SRR27199003	5,392,356	64	56.88	22	478,496	5,119	100
HKp16	Human	*K. pneumoniae sensu strico*	SAMN38811464	JAXSCK000000000	SRR27199002	5,565,637	69	55.79	32	508,313	5,392	99
HKp17	Human	*K. pneumoniae sensu strico*	SAMN38811465	JAXSCJ000000000	SRR27199001	5,547,533	80	56.24	36	391,789	5,396	96
HKp18	Human	*K. pneumoniae sensu strico*	SAMN38811466	JAXSCI000000000	SRR27199000	5,469,254	61	56.6	32	340,162	5,272	94
HKp19	Human	*K. pneumoniae sensu strico*	SAMN38811467	JAXSCH000000000	SRR27198998	5,521,504	32	56.4	41	353,306	5,341	95
HKp2	Human	*K. pneumoniae sensu strico*	SAMN38811468	JAXSCG000000000	SRR27198933	5,486,293	41	56.01	56	352,022	5,261	88
HKp20	Human	*K. pneumoniae sensu strico*	SAMN38811469	JAXSCF000000000	SRR27198932	5,492,507	34	56.19	59	255,498	5,267	83
HKp21	Human	*K. quasipneumoniae* subsp. *similipneumoniae*	SAMN38811470	JAXSCE000000000	SRR27198931	5,099,859	45	57.68	19	591,100	4,827	93
HKp22	Human	*K. pneumoniae sensu strico*	SAMN38811471	JAXSCD000000000	SRR27198930	5,524,670	69	55.69	53	288,852	5,327	96
HKp23	Human	*K. pneumoniae sensu strico*	SAMN38811472	JAXSCC000000000	SRR27198929	5,347,731	45	56.67	43	251,342	5,144	95
HKp24	Human	*K. pneumoniae sensu strico*	SAMN38811473	JAXSCB000000000	SRR27198928	5,405,787	49	56.24	60	216,924	5,342	98
HKp25	Human	*K. variicola* subsp. *variicola*	SAMN38811474	JAXSCA000000000	SRR27198927	5,356,039	43	57.19	20	482,246	5,106	95
HKp26	Human	*K. variicola* subsp. *variicola*	SAMN38811475	JAXSBZ000000000	SRR27198926	5,496,742	23	56.91	67	141,189	5,248	87
HKp28	Human	*K. variicola* subsp. *variicola*	SAMN38811476	JAXSBY000000000	SRR27198925	5,487,839	47	56.79	27	440,092	5,225	88
HKp29	Human	*K. pneumoniae sensu strico*	SAMN38811477	JAXSBX000000000	SRR27198923	5,773,613	51	56.34	38	356,169	5,628	94
HKp3	Human	*K. quasipneumoniae* subsp. *similipneumoniae*	SAMN38811478	JAXSBW000000000	SRR27198922	5,231,235	45	57.15	23	393,992	5,006	89
HKp30	Human	*K. pneumoniae sensu strico*	SAMN38811479	JAXSBV000000000	SRR27198921	5,526,729	68	56.03	49	289,601	5,322	98
HKp31	Human	*K. pneumoniae sensu strico*	SAMN38811480	JAXSBU000000000	SRR27198920	5,464,356	50	56.76	40	350,419	5,277	89
HKp32	Human	*K. pneumoniae sensu strico*	SAMN38811481	JAXSBT000000000	SRR27198919	5,563,033	44	56.71	39	282,176	5,361	96
HKp33	Human	*K. variicola* subsp. *variicola*	SAMN38811482	JAXSBS000000000	SRR27198918	5,492,619	41	56.91	29	440,646	5,229	91
HKp34	Human	*K. pneumoniae sensu strico*	SAMN38811483	JAXSBR000000000	SRR27198917	5,191,039	59	57.42	23	433,863	4,987	91
HKp35	Human	*K. pneumoniae sensu strico*	SAMN38811484	JAXSBQ000000000	SRR27198916	5,300,781	30	56.18	91	157,144	5,147	85
HKp36	Human	*K. pneumoniae sensu strico*	SAMN38811485	JAXSBP000000000	SRR27198915	5,495,317	45	56.98	46	350,109	5,280	95
HKp37	Human	*K. pneumoniae sensu strico*	SAMN38811486	JAXSBO000000000	SRR27198914	5,365,006	49	56.81	34	333,153	5,187	95
HKp38	Human	*K. pneumoniae sensu strico*	SAMN38811487	JAXSBN000000000	SRR27199058	5,579,148	38	55.77	99	129,962	5,420	97
HKp39	Human	*K. pneumoniae sensu strico*	SAMN38811488	JAXSBM000000000	SRR27199057	5,483,494	113	56.43	68	265,340	5,332	88
HKp4	Human	*K. pneumoniae sensu strico*	SAMN38811489	JAXSBL000000000	SRR27199056	5,437,624	45	55.98	131	95,856	5,330	94
HKp40	Human	*K. pneumoniae sensu strico*	SAMN38811490	JAXSBK000000000	SRR27199055	5,605,006	51	56.43	82	305,637	5,519	100
HKp41	Human	*K. pneumoniae sensu strico*	SAMN38811491	JAXSBJ000000000	SRR27199054	5,422,742	42	56.5	55	301,240	5,336	92
HKp42	Human	*K. variicola* subsp. *variicola*	SAMN38811492	JAXSBI000000000	SRR27199053	5,616,842	102	56.88	35	318,729	5,407	93
HKp43	Human	*K. pneumoniae sensu strico*	SAMN38811493	JAXSBH000000000	SRR27199052	5,350,440	49	56.93	25	356,104	5,140	87
HKp44	Human	*K. pneumoniae sensu strico*	SAMN38811494	JAXSBG000000000	SRR27199051	5,247,822	45	57.25	22	439,392	5,049	95
HKp45	Human	*K. pneumoniae sensu strico*	SAMN38811495	JAXSBF000000000	SRR27199050	5,334,273	51	56.82	47	323,114	5,136	90
HKp46	Human	*K. variicola* subsp. *variicola*	SAMN38811496	JAXSBE000000000	SRR27199049	5,506,348	57	57.22	21	404,218	5,230	90
HKp47	Human	*K. quasipneumoniae* subsp. *similipneumoniae*	SAMN38811497	JAXSBD000000000	SRR27199047	5,577,170	35	56.75	95	164,232	5,459	92
HKp48	Human	*K. pneumoniae sensu strico*	SAMN38811498	JAXSBC000000000	SRR27199046	5,603,980	66	56.4	68	326,559	5,514	99
HKp49	Human	*K. pneumoniae sensu strico*	SAMN38811499	JAXSBB000000000	SRR27199045	5,227,155	63	56.75	60	194,543	5,076	89
HKp5	Human	*K. pneumoniae sensu strico*	SAMN38811500	JAXSBA000000000	SRR27199044	5,197,967	84	56.61	39	392,908	4,966	101
HKp50	Human	*K. pneumoniae sensu strico*	SAMN38811501	JAXSAZ000000000	SRR27199043	5,525,194	64	56.83	61	266,427	5,410	95
HKp51	Human	*K. pneumoniae sensu strico*	SAMN38811502	JAXSAY000000000	SRR27199042	5,415,873	32	56.82	40	378,459	5,223	99
HKp52	Human	*K. variicola* subsp. *variicola*	SAMN38811503	JAXSAX000000000	SRR27199041	5,417,958	56	57.23	15	1,005,355	5,150	97
HKp53	Human	*K. pneumoniae sensu strico*	SAMN38811504	JAXSAW000000000	SRR27199040	5,473,219	62	56.98	26	581,574	5,272	94
HKp54	Human	*K. pneumoniae sensu strico*	SAMN38811505	JAXSAV000000000	SRR27199039	5,293,626	38	56.69	66	246,509	5,163	94
HKp55	Human	*K. pneumoniae sensu strico*	SAMN38811506	JAXSAU000000000	SRR27199038	5,490,231	41	56.46	55	371,210	5,265	91
HKp56	Human	*K. pneumoniae sensu strico*	SAMN38811507	JAXSAT000000000	SRR27199036	5,333,990	49	56.93	50	269,909	5,139	92
HKp57	Human	*K. variicola subsp. variicola*	SAMN38811508	JAXSAS000000000	SRR27199035	5,507,063	61	57.17	18	462,100	5,228	89
HKp58	Human	*K. pneumoniae sensu strico*	SAMN38811509	JAXSAR000000000	SRR27199034	5,387,393	52	56.68	39	333,445	5,229	94
HKp59	Human	*K. pneumoniae sensu strico*	SAMN38811510	JAXSAQ000000000	SRR27199033	5,607,908	88	56.31	74	326,559	5,521	99
HKp6	Human	*K. pneumoniae sensu strico*	SAMN38811511	JAXSAP000000000	SRR27199032	5,303,782	50	56.06	78	252,845	5,127	87
HKp60	Human	*K. pneumoniae sensu strico*	SAMN38811512	JAXSAO000000000	SRR27199031	5,511,978	44	56.6	58	295,498	5,389	92
HKp61	Human	*K. pneumoniae sensu strico*	SAMN38811513	JAXSAN000000000	SRR27199030	5,707,669	53	56.17	82	210,766	5,610	98
HKp62	Human	*K. pneumoniae sensu strico*	SAMN38811514	JAXSAM000000000	SRR27198997	5,783,949	42	56.1	49	262,494	5,655	93
HKp63	Human	*K. pneumoniae sensu strico*	SAMN38811515	JAXSAL000000000	SRR27198996	5,530,438	48	56.7	34	391,677	5,346	98
HKp64	Human	*K. pneumoniae sensu strico*	SAMN38811516	JAXSAK000000000	SRR27198995	5,349,402	67	56.99	25	353,558	5,135	90
HKp65	Human	*K. pneumoniae sensu strico*	SAMN38811517	JAXSAJ000000000	SRR27198993	5,426,112	96	56.02	67	343,360	5,272	94
HKp66	Human	*K. pneumoniae sensu strico*	SAMN38811518	JAXSAI000000000	SRR27198992	5,392,351	30	56.8	47	379,271	5,190	98
HKp67	Human	*K. pneumoniae sensu strico*	SAMN38811519	JAXSAH000000000	SRR27198991	5,192,727	56	56.77	33	403,964	4,958	94
HKp68	Human	*K. pneumoniae sensu strico*	SAMN38811520	JAXSAG000000000	SRR27198990	5,534,022	30	56.42	80	218,950	5,419	91
HKp69	Human	*K. pneumoniae sensu strico*	SAMN38811521	JAXSAF000000000	SRR27198989	5,618,659	77	56.37	54	336,934	5,454	97
HKp7	Human	*K. pneumoniae sensu strico*	SAMN38811522	JAXSAE000000000	SRR27198988	5,278,397	45	56.79	62	289,518	5,076	93
HKp70	Human	*K. quasipneumoniae* subsp. *quasipneumoniae*	SAMN38811523	JAXSAD000000000	SRR27198987	5,480,726	49	57.24	27	493,923	5,260	98
HKp72	Human	*K. pneumoniae sensu strico*	SAMN38811524	JAXSAC000000000	SRR27198986	5,494,696	54	56.13	53	321,085	5,265	90
HKp73	Human	*K. pneumoniae sensu strico*	SAMN38811525	JAXSAB000000000	SRR27198985	5,192,637	51	56.68	33	408,428	4,955	93
HKp74	Human	*K. variicola* subsp. *variicola*	SAMN38811526	JAXSAA000000000	SRR27198984	5,595,544	52	56.99	29	470,508	5,390	95
HKp75	Human	*K. pneumoniae sensu strico*	SAMN38811527	JAXRZZ000000000	SRR27198982	5,193,738	45	56.82	30	408,428	4,954	94
HKp76	Human	*K. pneumoniae sensu strico*	SAMN38811528	JAXRZY000000000	SRR27198981	5,559,252	56	56.48	48	334,965	5,461	97
HKp77	Human	*K. pneumoniae sensu strico*	SAMN38811529	JAXRZX000000000	SRR27198980	5,548,795	30	56.55	57	304,407	5,460	93
HKp78	Human	*K. pneumoniae sensu strico*	SAMN38811530	JAXRZW000000000	SRR27198979	5,407,764	78	56.68	37	304,230	5,205	91
HKp79	Human	*K. pneumoniae sensu strico*	SAMN38811531	JAXRZV000000000	SRR27198978	5,408,561	44	56.54	37	305,094	5,207	90
HKp8	Human	*K. pneumoniae sensu strico*	SAMN38811532	JAXRZU000000000	SRR27198977	5,382,904	59	56.67	39	333,445	5,222	93
HKp80	Human	*K. pneumoniae sensu strico*	SAMN38811533	JAXRZT000000000	SRR27198976	5,405,426	47	56.57	39	265,416	5,205	91
HKp9	Human	*K. pneumoniae sensu strico*	SAMN38811534	JAXRZS000000000	SRR27198975	5,496,703	32	56.55	49	311,929	5,291	89

^*a*^ "SRA”,Seqeunce Read Archive.

^*b*^ "CDS”,Coding Sequence.

## Data Availability

Raw sequencing reads corresponding to each sample are deposited under BioProject Accession number PRJNA1052047.
